# Application of a high-quality, high-volume trap–neuter–return model of community cats in Seoul, Korea

**DOI:** 10.7717/peerj.8711

**Published:** 2020-03-05

**Authors:** Yoonju Cho, Kyunghee Kim, Min Su Kim, Inhyung Lee

**Affiliations:** 1 Department of Pet Science, Seojeong University, Yangju, Gyeonggi, Republic of Korea; 2 Gyeongin Animal Medical Center, Seoul, Republic of Korea; 3 Department of Veterinary Clinical Sciences, College of Veterinary Medicine and Research Institute for Veterinary Science, Seoul National University, Seoul, South Korea

**Keywords:** TNR, Feral cat, Population control, Urban environment, Shelter, HQHVSN clinics, Neutering, Sterilization

## Abstract

**Background:**

This study was performed to determine the characteristics of community cats that were admitted to trap–neuter–return****(TNR) programs and the feasibility of the high-quality, high-volume spay/neuter (HQHVSN) model in Seoul, Korea.

**Methods:**

TNR programs were performed eight times from 2017 to 2018, and a total of 375 community cats from the pilot areas were admitted. The pilot areas were selected regions wherein caregivers voluntarily participated in our TNR program. Each cat was anesthetized, assessed for health status, sterilized, vaccinated against feline viral rhinotracheitis, calicivirus and panleukopenia (FVR-CP), and rabies, and treated with insecticide after surgery. The time from anesthesia to recovery was evaluated to verify the efficiency of surgical time of the program. The TNR program at a local animal hospital and the program in this study were compared to assess the veterinary treatment administered and the cost for each cat.

**Results:**

A total of 375 cats were underwent TNR in this study, including 192 (51.2%) intact females, 180 (48%) intact males, and three (0.8%) sterilized cats. Following surgery, 372 cats (99.2%) were returned to their original locations. Three cats (0.8%) died postoperatively. On average, 21.9% of the cats were pregnant during the TNRs, and the highest percentage of cats (63.9%) were pregnant in March. All cats presented to the TNR program were considered healthy enough to be part of the program after examination. For neutering one animal at a time, similar to conventional TNR ($140), it took 53 ± 16 mins for females and 30 ± 9 mins for males from anesthesia to recovery. In contrast, the standardized procedure can neuter multiple cats simultaneously, similar to the conveyor system, at an estimated rate of 6.8 mins per cat ($45 per female cat, $30 per male cat).

**Conclusions:**

The TNR strategy in Seoul should be implemented by establishing dedicated clinics to concentrate on HQHVSN surgeries of cats. Through this pilot study, we were able to demonstrate that it is possible to effectively apply HQHVSN clinics in Korea. Future studies that perform intensive sterilization in targeted areas are necessary to confirm the efficacy of the TNR strategy.

## Introduction

According to the Korean Animal Welfare Act, community cats that naturally live in urban or residential areas should not be admitted to the animal shelter. Instead, measures are taken to neuter them to control the population and release them to capture sites. However, according to statistics from the Animal Protection Management System (APMS), kittens and injured cats from the community are the greatest source of feline intake at Korea’s animal shelters (APMS, 2018; Cho et al., 2015). These free-roaming, unowned stray and feral community cats in Korea are called ‘street cats’. The neologism, “cat mom,” refers to people who voluntarily feed or take care of street cats. Recently, an increase in community cat populations started causing problems, including community cat welfare issues, nuisance behaviors, and public health concerns (Kilgour et al., 2017; South Korean Ministry of agriculture, food and rural affairs, 2018; Van et al., 2019). To reduce the population of community cats, a humane population control strategy known as trap–neuter–return (TNR) was introduced in 2008. The number of community cats in Seoul is estimated to be approximately 140,000 (Seoul Metropolitan Government, 2019). Between 2016 and 2018, on an average, only 10,000 cats were sterilized. Many studies have shown that population control via TNR is effective in reducing the number of cats and nuisance concerns over time (Centonze & Levy, 2002; Finkler, Gunther & Terkel, 2011; Levy, Isaza & Scott, 2014; Spehar & Wolf, 2018; Swarbrick & Rand, 2018). TNR at the colony level alone has been insufficient in reducing the population of community cats and reducing the number of cats admitted to animal shelter (Spehar & Wolf, 2018). The Korean municipality commissions some local animal hospitals to implement sporadic TNR and pays them about $140 per cat (South Korean Ministry of agriculture, food and rural affairs, 2018). Because of the limited resources allocated, the number of cats that can be neutered is limited; the number of cats that can be neutered is pre-estimated when calculating a given budget. This amount includes the cost of capture, release, and the cost of surgery, excluding vaccination and deworming. Therefore, it is necessary to modify the strategy to sterilize more cats within the limited budget. Several countries have modified the TNR strategy to focus on specific areas with larger feline populations, and implemented intensive sterilization with the goal of expanding TNR programs into surrounding areas (Nutter, 2006; Gunther, Finkler & Terkel, 2011; Mendes-de Almeida et al., 2011; Johnson & Cicirelli, 2014; Spehar & Wolf, 2018; Zito et al., 2018). This targeted, high-impact TNR intervention is more effective than sporadic TNR in reducing the community cat population (Kortis, 2014; Levy, Isaza & Scott, 2014; Kilgour et al., 2017). However, there are no high-quality, high-volume spay-neuter (HQHVSN) clinics in Korea, and there is a lack of experience in adopting high-impact TNR methods. To improve the TNR programs for the control of community cats in Korea, a HQHVSN clinic model has been used since February 2017. In the current study, we adopted the Operation Catnip program, a HQHVSN program conducted at the College of Veterinary Medicine, University of Florida. Trained volunteers, including veterinarians, veterinary students, and veterinary technicians, were assigned to each station.

According to the data reported by the Seoul government, a cumulative total of 56,266 community cats were sterilized from 2012 to 2018. Nevertheless, to date, there has been no collection of information on the characteristics of community cats admitted to TNR programs; therefore, we attempted to gather data on the health status and basic information of community cats in Seoul in the current study. In addition, this study aims to introduce a TNR strategy for the first time in Korea using the HQHVSN system that can safely and simultaneously neuter a large number of community cats. This data will be important for developing better strategies to control the population of community cats in Seoul and other parts of Korea.

## Material and Methods

### Selected pilot area

Seoul is the capital of Korea and is highly urbanized. It has an estimated population of 10,124,579 people (Government Seoul Metropolitan, 2017). Seoul is divided into 25 administrative districts, and each borough has a caregiver council to present its opinions on community cat management. The boroughs selected as the pilot area were regions in which caregivers voluntarily participated in our TNR program. Caregivers who cared for intact community cats agreed to trap and return cats for neutering. Cats were sterilized in a total of eight high-quality, high-volume TNR programs from 2017 to 2018.

### Trap and return

Since Korea does not have a dedicated place to perform a HQHVSN study, a temporary operation site was set up using an empty building owned by the Seoul Metropolitan Government. Additionally, the government provided vehicles to efficiently transport the cats to the temporary HQHVSN clinic.

Because of limitations in the temporary operating area, a program was set up to sterilize 50 community cats at a time. In advance, the caregivers were asked to capture up to 50 cats. Caregivers performed the trapping with manual or automatic cage traps and post-operative releasing of community cats (mostly feral cats). The cats were contained in traps for 24 h (male) and 72 h (female) after surgery and then released back to their colonies. Sterilized cats were not given post-operative analgesia and were checked for food intake, urination, and defecation during the holding period in traps.

### Neuter

At the temporary clinic, each cat passed through a series of treatment stations. Anesthetists assessed the general health and appearance of the cats and screened them for any potential contraindications for anesthesia. Each cat was anaesthetized with an intramuscular injection (0.5 ml) of tiletamine-zolazepam (19 mg/cat, Zoletil50; Virbac), ketamine (23 mg/cat, Ketamine; Yuhan), and xylazine (3.8 mg/cat, Xyzine; SF. INC) mixture through the wire mesh of a humane trap while the cat remained in the trap (Williams et al., 2002). After induction of anesthesia, veterinarians performed a physical examination to identify any conditions that required additional treatment and recorded the medical record of each cat on the air cap bubble wrap, which was used to maintain the body temperature. After physical examination, animals deemed healthy enough for surgery had the tip of their left ear ( one cm of the tip) removed for visual identification and were administrated intramuscular injections of penicillin G + dihydrostreptomycin sulphate (25,000 IU/kg, PPS; Daesung Microbiological Labs) and subcutaneous injections of meloxicam (0.3 mg/kg, Metacam; Boehringer Ingelheim). The surgical area was shaved and scrubbed with chlorhexidine (Alphahexidine; Firson). In every TNR program, five to six veterinarians operated on female cats and one veterinarian operated on male cats. Neutered cats were vaccinated against feline viral rhinotracheitis, calicivirus and panleukopenia (FVR-CP, Nobivac Tricat Trio; MSD Animal Health), and rabies (Nobivac Rabies; MSD Animal Health) in the left and right hind legs, respectively. Topical parasite control (imidacloprid and moxidectin, Advocate; Bayer) was applied to the skin at the base of the skull and yohimbine (0.1 mg/kg IV, Zyverse; SF. INC) was administered to reverse the effect of xylazine. Some cats were subcutaneously administered normal-saline fluids or cefovecin sodium (8 mg/kg, Convenia; Zoetis) as ordered by a veterinarian. Animal care was approved by the Seojeong University Institutional Animal Care and Use Committee (SJ2018-03).

### Data analysis

The TNR program performed in local animal hospitals and that performed in this study were compared to assess the veterinary treatments and cost per cat. Data from the TNR program performed in local animal hospitals was collected and analyzed retrospectively (South Korean Ministry of agriculture, food and rural affairs, 2018). The information for each cat undergoing surgical neutering was completed by a veterinarian (local veterinarian or graduate veterinarian). Data recorded included maturity, sex, vital signs, medical conditions, pregnancy status, number of fetuses, and the list of medications administered to the animals. Necropsy was not performed on cats that died. Results are presented as mean ± SD (range) or percentage of animals. This comparison was performed with the Chi-square test and the independent *t*-test.

## Results

A total of 375 cats were admitted to the eight TNR programs over the two-year study period (150 in Year 1 and 225 in Year 2). This included 192 (51.2%) intact females, 180 (48%) intact males, and three (0.8%) previously sterilized cats. A total of 372 cats (99.2%) were returned to their original locations. Three female cats (0.8%) died during the TNR programs; two of which were pregnant, the other was nursing (Table 1).

**Table 1:** Characteristics of 375 community cats admitted to eight trap-neuter-return programs in Seoul.

	**Total**	2017				2018			
		Feb	Sep	Oct	Nov	Feb	Mar	Sep	Oct
Intact females	192 (51.2%)	19 (44.2%)	11 (33.3%)	26 (59.1%)	19 (63.3%)	21 (50.0%)	36 (63.2%)	22 (51.2%)	38 (45.8%)
Intact males	180 (48.0%)	24 (55.8%)	22 (66.7%)	18 (40.9%)	11 (36.7%)	20 (47.6%)	21 (36.8%)	20 (46.5%)	44 (53.0%)
Previously sterilized	3 (0.8%)	0	0	0	0	1 (2.4%)	0	1 (2.3%)	1 (1.2%)
Pregnant females	42 (21.9%)	6 (31.6%)	2 (18.2%)	1 (3.8%)	0	7 (33.3%)	23 (63.9%)	3 (13.6%)	0
Foetuses per litter^a^	4.4 ± 0.25	5.5 ± 1.4	4.0 ± 0.0	3.0 ± 0.0	0	4.6 ± 0.9	4.0 ± 1.7	5.3 ± 1.9	0
Nursing females	19 (9.9%)	0	2 (18.2%)	4 (15.4%)	2 (10.5%)	1 (4.8%)	0	7 (31.8%)	3 (7.9%)
Died	3 (0.8%)	0	0	1 (2.3%)	0	0	1 (1.8%)	1 (2.3%)	0

**Notes.**

a Mean ± standard deviation.

Overall, 21.9% of female cats (*n* = 42) were pregnant at the time of surgery (Table 1), and the highest percentage of cats (63.9%) were pregnant in March. The mean number of fetuses per litter was 4.4 ± 0.25 (range, 2–8 fetuses). At the time of surgery, 9.9% of female cats (*n* = 19) were nursing. Cryptorchidism was not observed in any male cat admitted for sterilization.

The average body weight of female and male cats was 3.30 ±0.84 kg and 3.78 ± 1.17 kg, respectively. They were checked for any abnormalities in the eyes, nose, ear, mouth and skin, their heart rate, temperature, respiratory rate, or mucous membrane color. None of the cats required immediate intervention, and there were no significant findings to postpone surgery. Therefore, all cats admitted to the TNR program were neutered. However, 28.3% of the cats had gingivitis, 5.6% had mild to severe stomatitis, and 26.4% had brown debris in their ear canals. The cause of the brown debris was not determined, and it was wiped off with an ear cleaner. The cats with gingivitis or stomatitis in their mouths weighed significantly less than the cats that did not (*t*_(373)_ =  − 2.525, *p* = 0.012). In addition, 10.1% of the cats had skin injuries. *T*-test results showed that males had significantly more skin injuries than their female counterparts (Chi^2^_(1,375)_ = 6.879, *p* = 0.009). Unidentified discharge was observed in 4% of the cats’ noses.

The average individual surgery time required for recovery after anesthesia was 53 ± 16 mins and 30 ± 9 mins for female and male cats, respectively. The total operation time (from when the first cat was anaesthetized for each project to the last cat being recovered, regardless of sex) for the 372 cats was 42 h and 24 mins. This indicated that each operation took 6.8 mins per cat, thus implying that the present method had more efficient surgical time compared to the traditional TNR method.

The veterinary treatments and costs are presented in Table 2. The TNR program performed in local animal hospitals does not include the FVRCP vaccination and parasite control treatment administered to community cats. In addition, for community cats that needed personalized treatment, additional expenses are paid at the caregivers’ discretion. Rabies vaccine is recommended by the local government, but history of vaccination could not be confirmed because of the unavailability of vaccination records. In the HQHVSN system, the costs ($30 per male cat, $45 per female cat) for neutering are lower than that of the traditional method ($140) because the cost for other procedures, except the material costs, are borne by volunteers.

**Table 2:** Veterinary treatments and cost performed on community cats treated at each trap-neuter-return program in Seoul.

	TNR in local animal hospital	HQHVSN model
Spay and Neuter	Yes	Yes
Ear tipped	Yes	Yes
FVRCP vaccination	No	Yes
Rabies vaccination	Optional	Yes
Parasite control	No	Yes
Long-term antibiotic	No	Optional
Record keeping	Body weight, color, age, body condition	Body weight, color, age, physical examination check list, medical record, surgical record
Postoperative care	Staff in local animal hospital	Veterinary volunteer and Caregivers
Trap and Return	Charging	Volunteering
Surgical neutering	$140/ per cat	$45 (female), $30 (male)

**Notes.**

FVRCP: feline herpes virus, feline calicivirus, feline panleukopenia virus; Long-term antibiotic: cefovecin (Convenia^®^, Administrations in local animal hospital are charged to the caregivers who requested them).

## Discussion

We collected information on the health status of the trapped cats and evaluated the time and cost efficiency of mass sterilization of cats using the HQHVSN system. However, the guidelines for the TNR of the community cat, which were created by the Ministry of Agriculture and Forestry, prohibits the sterilization of pregnant or lactating females (Ministry of agriculture, food and rural affairs, 2016). This guideline has been established in 2016 with the advice of animal rights groups and some veterinarians, in respect for the life of the fetus. The guidelines also state that surgery can be performed if veterinarians become aware of the pregnancy only after the laparotomy is performed. In this TNR program, sterilization surgeries were performed in pregnant and lactating subjects with prior approval from Seoul City officials, caregivers, and IACUC. Because a pregnant cat’s uterus is removed at once without dissection, the fetus would not experience consciousness regardless of the stage of pregnancy and will die without pain. (White, Jefferson & Levy, 2010; Leary et al., 2013)

In the current study, the average prenatal litter size was 4.4 ± 0.25 fetuses per litter (range, 2–8). Whereas the average of number of fetuses per litter in 16,190 pregnant cats was only 4.1 ± 0.1 fetuses in the United States (Wallace & Levy, 2006). The trapped cats were fed directly by a caregiver in this study. Abundant food resources increase the fecundity of female cats and also increase the survival of kittens (Schmidt, Lopez & Collier, 2007). Previous studies have shown that cats in urban areas have decreased range of habitation and increased population density due to abundantly available food resources (Horn et al., 2011; Thomas, Baker & Fellowes, 2014). The wild felids, including feral cats, are solitary, but community cats have been identified as a separate group because they are able to obtain stable food resources (Liberg & Sandell, 2000). Sustainable access to food increases the survival and fecundity of cats and reduces their range and overall movement (Schmidt, Lopez & Collier, 2007); therefore, the density of cats increases when the availability of food is high. This increases the number of complaints associated with cats and leads to the transmission of diseases and the predation of wildlife.

Through this intensive TNR, it was possible to sterilize 42 pregnant cats and prevent the birth of 185 kittens. Korean municipal animal shelters house a large number of feral kittens in the kitten season (May to October), and the total survival rate of cats is only 30.5% (Cho et al., 2015). In addition, about 75% of the feral kittens in urban areas die or disappear 6 months after birth because of various reasons, despite daily provision of food and water (Nutter, Levine & Stoskopf, 2004). Therefore, the sterilization of pregnant female cats may ultimately improve the welfare of feral cats.

Male cats (*n* = 26) had significantly more skin wounds than females (*n* = 12). However, after surgical neutering, fighting between males reduces and the risk of acquiring infectious diseases from wounds sustained during fights can reduce. In addition, neutering is expected to reduce noise disturbance by reducing the vocalization that occurs when fighting (Finkler, Gunther & Terkel, 2011). The severity of the gingivitis and stomatitis was not recorded when present, but cats with dental disease weighed significantly less than normal cats. Cats with dental disease were, in general, in good body condition. However, because this condition is somewhat common and can severely impact quality of life, it seems necessary to have equipment and veterinary expertise to perform dental extractions during TNR programs.

Since our TNR program was implemented on a temporary basis, it was difficult to determine the monthly population characteristics of cats in Seoul. However, the figures are very similar to the monthly pregnancy rate of female cats in previous studies (Wallace & Levy, 2006). In particular, 63.9% of female cats were pregnant in March. Additionally, to prohibit the sterilization of pregnant and lactating cats, guidelines state that operations should not be conducted in severe hot, rainy, or cold seasons (Ministry of agriculture, food and rural affairs, 2016). Korea has seasonal weather (i.e., spring, summer, fall, and winter). Midsummer ranges from July to August, and winter ranges from December to February. The guidelines have not been actively promoting TNR during these periods. Since cats are pregnant from March to April and lactating from May to June, TNR cannot be performed during these months. Between March and June, although the temperature is appropriate for trapping and releasing, TNR cannot be implemented because of the breeding season of cats, i.e., cats are generally pregnant or nursing. Therefore, there is a limited amount of time to actively perform TNR. In this TNR program, caregivers were asked to return lactating cats to the area where they were found as soon as possible (24 h postoperatively).

Also, the guidelines prohibit surgery on cats under 2 kg (Ministry of agriculture, food and rural affairs, 2016). There is a misconception that small cats may be harassed by other adult cats and that they may require a longer recovery time after undergoing surgery. However, the surgical procedures for young adult cats are easier, faster, and safer than the procedures used for older cats (Kustritz, 2007; Bushby & Griffin, 2011; Porters et al., 2014; Griffin et al., 2016). Indeed, probability of disease (mammary hyperplasia, ovarian cysts, and increased friability and vascularity of the uterus) in younger female cats is lower than in older female cats, and postoperative recovery is faster in younger cats than in older cats (Yates & White, 2018). Cats begin their first estrous cycle at 5 to 9 months of age, but estrus can be accelerated if their nutritional status and habitat are stable (Feldman & Nelson, 2004). In addition, it is known that estrus can be accelerated if females are exposed to tom cats in the surrounding area. Further, under appropriate physical and nutritional conditions, females can become pregnant when they reach a weight of approximately 2 kg (Little, 2011). If female cats that are less than 2 kg were released without surgery, it is likely that they will become more fertile and compensate for the lack of breeding in areas where a TNR strategy is implemented (Gunther, Finkler & Terkel, 2011). Although various studies have investigated the use of non-surgical contraceptive methods, such as administration of the gonadotropin-releasing hormone (GnRH) vaccine, which have relatively fewer restrictions with regards to the weather or age of cats, it is not yet a feasible alternative to surgery (Levy, 2011; Levy et al., 2011; Fischer et al., 2018; Lee et al., 2019).

In the current study, unexpected deaths occurred in three (0.8%) cats during the recovery process. The exact cause of death could not be determined. However, two of these cats were pregnant, and are suspected to have excessively lowered body temperature due to prolonged surgical time and delayed recovery due to an anesthetic overdose in comparison to their body weight. This figure is slightly higher than other TNR programs where 0.2% to 0.7% of the animals had died (Wallace & Levy, 2006; Williams et al., 2002). To prevent fatal complications, facilities must be properly equipped to perform anesthesia, monitor patients, and deal with emergencies. Above all, dedicated spay/neuter clinics to perform high-quality surgeries are required (Frank & Carlisle-Frank, 2007).

The Seoul Animal Protection Division was founded in 2012. To reduce the intake of community kittens in the shelter, the Animal Protection Division restricted the rescue targets to only kittens that were abandoned by their mother. At the same time, the TNR program has also been expanded to neuter a larger number of community cats. Although the number of community cats sterilized has increased since 2013, the intake of community cats at shelters has not decreased significantly. Since the number of cats in the city is approximately 140,000 (Seoul Metropolitan Government, 2019), fewer than 7% of them are neutered every year. As more cats need to be neutered, we cannot rely on animal hospitals alone to drastically increase the number of TNRs. Local animal hospitals perform a limited number of surgeries per day and are more expensive than the HQHVSN program. This traditional TNR program charges $140 for sterilizing one cat and has been practiced for 10 years, with a slight increase in cost during that period. However, it was difficult to confirm the rationale for calculating this amount. Within these costs, the animal hospital pays the fee by entrusting it with the person in charge of trap and return. The amount varies with hospital, but is about $50. These intermittent TNR programs cost about $45 for one female cat and $30 for one male cat, as all personnel volunteered for implementation. This cost is much lower than that required with the traditional method, which costs $140 per cat, and moreover, includes additional benefits (vaccination, deworming, etc.). Therefore, opening non-profit HQHVSN clinics would be a more realistic alternative although the cost to start-up is rather high.

This program was conducted to introduce the HQHVSN model for targeted TNR in Seoul. This study was limited to 8 rounds of temporary TNR programs over 2 years, and could not confirm the monthly status of community cats in Seoul. The cats trapped in this study were fed directly by the caregiver and they were provided with good nutrition; therefore, their health is not representative of the entire community cat population in Seoul. In addition, since the trap sites could not be documented by zip code, it could not trace the changes in the number of cats after TNR. However, it was important that the volunteers and veterinarians experienced the positive effects of the systematic HQHVSN models and shared the need for public facilities to operate on a large number of community cats. Currently, sporadic TNR and colony-TNR are known to be insufficient to reduce the number of cats in certain areas (White, Jefferson & Levy, 2010; Spehar & Wolf, 2018). It has not been possible to confirm the impact of TNR performed in Korea because there are no published analyses of local neuter programs. More prospective studies should be conducted to determine the effectiveness of various strategies to control the population of community cats in Korea.

## Conclusion

Recently, the local government planned to capture more community cats and perform safe sterilization. This pilot project demonstrated that a mass neutering program for community cats is feasible in Korea. It is necessary to create public facilities to increase the number of TNR programs and to change the guidelines that currently place many restrictions on TNR. The ban on surgery for cats weighing less than 2 kg and pregnant or lactating cats should be relaxed. Specific guidelines on the weather conditions at the time of trap and return must be provided. Budgeting for each community cat and neutering in a private veterinary clinic is limited in controlling populations. To compensate for this, a variety of strategies should be tried to effectively and economically carry out TNR. The results from this study demonstrate the feasibility of a high-quality, high-volume TNR program for controlling the community cat population in Korea. Further characteristics of the community cat population collected from this study will provide the basis for population management policy of community cats in the future.

##  Supplemental Information

10.7717/peerj.8711/supp-1 Supplemental Information 1 Raw data

## Additional Information and Declarations

### Data Availability

The following information was supplied regarding data availability: The raw measurements are available in the Supplemental Files.
